# Inequity in the transmission of malaria infection among children and adolescents: a cohort study in rural Guinea

**DOI:** 10.1038/s41598-025-15600-w

**Published:** 2025-08-14

**Authors:** Almamy Amara Toure, Sidikiba Sidibe, Aboubacar Sidiki Magassouba, Abdoul Habib Beavogui, Mamoudou Conde, Abdoulaye Fodé Toure, Tiany Sidibe, Kaba Saran Keita, Alexandre Delamou, Seni Kouanda

**Affiliations:** 1 Institut Africain de Santé Publique (IASP/USTA) of the University Saint Thomas D’Aquin, Ouagadougou, Burkina Faso; 2 Department of Public Health, Faculty of Health Sciences and Techniques, Gamal Abdel Nasser University, Conakry, Guinea; 3 Centre National de Formation et de Recherche en Santé Rurale de Mafèrinyah, Forécariah, Guinea; 4 National Institute of Public Health, Coyah, Guinea; 5 Department of Public Health, Center for Research in Reproductive Health, Guinea, Conakry, Guinea; 6 Centre d’Excellence Africain pour la Prévention et le Contrôle des Maladies Transmissibles (CEA-PCMT), Gamal Abdel Nasser University, Conakry, Guinea; 7 Institut Africain de Santé publique, Ouagadougou, Burkina Faso

**Keywords:** Inequity, Malaria transmission, Children, Adolescents, Rural Guinea, Health care economics, Epidemiology

## Abstract

Malaria remains a major health concern in rural Guinea, where children face fivefold higher infection risk compared to urban areas. While disparities between rural and urban regions have been documented, inequities within rural areas remain unexplored. Socioeconomic factors influence malaria risk, which varies according to season. Understanding seasonal variations in socioeconomic factors is crucial for developing equitable and targeted interventions. This cohort study was conducted in Mafèrinyah, Guinea, with participants aged 1–19 years of age. Data were collected through monthly home visits over nine months, capturing malaria infection (via blood smears), sociodemographic factors, and household characteristics. Equity analysis was guided by the PROGRESS (place of residence, race/ethnicity/culture/language, occupation, gender/sex, religion, education, socioeconomic status, and social capital) framework. Statistical analyses included concentration index (CI) calculations to assess socioeconomic-related inequality across dry and rainy seasons, binary mixed-effects logistic, and decomposition of CI to identify contributing factors. Malaria infection varies according to season, age, and household characteristics. Children in households with older heads (50–77 years) had higher malaria odds during the dry season (OR = 3.44), whereas adolescents in the rainy season were more vulnerable in middle-aged-headed (OR = 15.78) and single-parent households (OR = 4.52). Concentration indices showed modest pro-rich inequity among adolescents in both seasons (CI = 0.059–0.085) and mixed patterns among children. Among children, secondary education (246.8%) and older age of household head yield strong malaria prevention dividends, but these benefits are currently captured by the better off, while primary education had strong pro-poor effects (among poorer households whose heads had only primary education). Among adolescents, middle-aged heads and older heads (− 419.3%), and unemployment (− 184.2%) reduced inequity. Male-headed households reversed roles by season, contributing − 67.2% to inequity in the dry season and + 19.5% in the rainy season. This study revealed significant seasonal and socioeconomic disparities in malaria transmission among children and adolescents in rural Guinea. Key equity drivers include the household head’s age, education, gender, and occupation, which have distinct seasonal effects. These findings highlight the need for targeted, equity-sensitive interventions that address structural vulnerabilities and seasonal dynamics to reduce the malaria burden and promote health equity.

**Supplementary Information:**

The online version contains supplementary material available at 10.1038/s41598-025-15600-w.

## Introduction

Malaria remains a major global public health challenge, disproportionately impacting children and adolescents. According to the latest malaria report focusing on addressing inequity in the global malaria response, there were an estimated 263 million cases and 597 000 malaria deaths worldwide in 2023, and approximately 95% of the deaths occurred in the WHO African Region, where many at risk still lack access to the services they need to prevent, detect, and treat the disease^1^. Globally, children and adolescents are disproportionately affected, with the disease responsible for over 75% of malaria-related deaths in children under five years of age^1^. Although efforts to scale up malaria control interventions have reduced the incidence in some regions, malaria continues to be shaped by structural inequities, particularly in rural areas with limited access to healthcare^2,3^, poor housing^4^, and inconsistent prevention coverage^5,6^.

Rural-urban disparities in malaria transmission have been well documented^6–11^, with rural populations consistently facing a higher prevalence^12–15^ and poorer access to prevention and treatment strategies^2,3^ than their urban counterparts. However, this binary framing often masks heterogeneity within rural settings. Increasing evidence has shown that intrarural inequity differences among rural households based on socioeconomic status^16–18^, occupation^19,20^, gender of the household head^20^, education^16,21^, and geographic location^22^ can significantly influence the risk of malaria. For instance, wealthier rural households may afford better housing and protection, whereas poorer households may be more exposed to vector habitats and face barriers to timely treatment^23^.

Moreover, malaria transmission is strongly influenced by seasonality, with transmission peaks aligned with rainfall patterns^24^, temperature^24^, and agricultural cycles^25^. However, few studies have investigated how seasonal variation modifies the patterns of social inequity in the malaria burden, particularly among younger populations, whose exposure and care dynamics differ by age group and household role. Seasonal changes in livelihood and time spent outdoors^26^ can exacerbate vulnerability, especially among rural households with constrained resources during dry or rainy seasons.

To date, little is known about how malaria-related health inequities unfold within rural communities and how these patterns vary by season despite clear shifts in vector ecology, household livelihoods, and healthcare access across the year.“In Guinea, malaria has high transmission rates in rural areas, where children are five times more likely to be infected than their urban counterparts^27^. These disparities reveal deep inequities exacerbated by socioeconomic factors and access to health care. Children from the poorest quintiles exhibit higher prevalence rates, highlighting the urgency of targeted interventions^27^. Understanding these inequities is essential for developing more equitable and effective control strategies aimed at reducing not only transmission but also severe consequences, such as severe anaemia, which affects up to 12% of chldren in the most disadvantaged households^27^.

This study aimed to explore the intra-rural inequities in malaria infection among children and adolescents in rural Guinea, specifically focusing on how seasonal variation modifies the role of social determinants such as socioeconomic status, household structure, gender, education, and geography.”

## Methods

### Study setting

This study was conducted in Maferinyah, a sub-prefecture in the Forécariah prefecture of Kindia, Republic of Guinea. Situated approximately 50 km from Conakry, Maferinyah lies between major urban centres and is undergoing a demographic shift from rural to semi-urban, making it ideal for examining intra-rural heterogeneity. Ecologically, the region’s dense vegetation and tropical, humid climate with a rainy season spanning May to October create highly favourable conditions for malaria transmission. Indeed, national surveys have documented malaria prevalence rates of approximately 32%^28^ among children in this region using microscopy. Maferinyah also benefits from a robust local infrastructure: it is home to the National Centre for Training and Research in Rural Health, which has conducted malaria research since 1995 and contributed to national malaria control strategies. Additionally, a community health training school and a medico-surgical centre provide essential services and enable effective collaboration with local health workers. These combined factors, high endemicity, demographic diversity, and institutional capacity, make Maferinyah a strategically valuable site for investigating intra-rural malaria transmission dynamics and social inequities.

### Study design

This study employed a cohort design with repeated measures and tracked participants from March 2022 to February 2023. Nine household visits were conducted during the 12-month period. Due to logistical constraints, two of these rounds were conducted as combined visits across consecutive months, specifically in June-July and October-November 2022. This allowed for consistent follow-up of the participants while adapting to field conditions.

### Study population and selection criteria

The study population included minors aged 1–9 years and adolescents aged 10–19 years residing in the Maferinyah sub-prefecture. The inclusion criteria were parental or legal guardian consent for minors and assent from adolescents aged 12–19 years. Households had to have at least three eligible residents, including at least one child aged under 18 years. Individuals with psychiatric illnesses or other conditions that could compromise their safety or rights were excluded from the study.

### Sampling method

Prior to the commencement of the study, a comprehensive census was conducted to enumerate all households in Maferinyah. This census enabled the creation of a household-based sampling frame, from which eligible households were randomly selected using probabilistic techniques to ensure representativeness. Within each selected household, all residents aged 1–19 years were screened for eligibility, and those who met the inclusion criteria, comprising both children (1–9) and adolescent (10–19 years), were invited to participate. Informed consent was obtained from the parents or guardians, and assent was obtained from the adolescents, as appropriate.

### Sample size justification

The required sample size was estimated based on prior studies reporting an incidence of 0.75 malaria episodes per person/year. To estimate this rate with a 95% confidence interval of ± 0.20 episodes per person per year and allowing for potential attrition and stratified analyses by age group, a total of 300 participants (children and adolescents) was deemed sufficient. The calculation was based on a Poisson distribution, which is suitable for modelling incidence rates in a cohort design.

### Study variables

#### Dependent variable

Malaria infection was defined as the presence of asexual plasmodium forms (ring-stage trophozoites) in peripheral blood smears with or without accompanying clinical symptoms. Gametocytes were recorded when observed but were not used to define the infection status, as they may persist beyond the resolution of active asexual parasitaemia.

#### Independent variables

- Sociodemographic data (age, residence, gender, educational level, and occupation).
- Socioeconomic factors (household assets as proxies).

### Data collection

The research team collected data using standardised questionnaires and clinical examinations by the research team. Biological tests included blood samples for malaria diagnosis and haemoglobin levels, as well as pregnancy tests for women of childbearing age. Socioeconomic and demographic information were collected, along with clinical data. All data were entered into the Open Data Kit (ODK) application and uploaded to the ONA platform for further analysis.

### Parasitological examination

Malaria diagnosis was conducted using both thick and thin blood smears stained with 10% Giemsa solution, following standard protocols. The thick smear was primarily used to detect and quantify Plasmodium parasites, with a detection threshold as low as 5–10 parasites per microlitre of blood, allowing the identification of low-density infections. A thin smear was used to identify the Plasmodium species and assess parasite morphology, including ring-stage trophozoites and gametocytes. All smears were examined under a binocular optical microscope by experienced microscopists to ensure accurate diagnosis and staging of the disease.

### Equity framework

This study employed the PROGRESS (Place of residence, Race/ethnicity, Occupation, Gender, Religion, Education, Socioeconomic status, and social capital) framework, which is widely used^29^, to explore the equity dimensions affecting malaria infection rates among children and adolescents in Maferinyah. In this study, we adopted a health equity lens to distinguish between inequality and inequity in the malaria burden. Inequality refers to observed, often quantifiable differences in health outcomes across socioeconomic groups, whereas inequity refers to systematic, avoidable, and unjust differences^30,31^. As outlined by the World Health Organization, not all health inequalities are inequities, but all health inequities manifest as inequalities^32^.

Given the rural setting, the analysis focused on intra-regional differences within sub-prefectures. We assessed disparities among various sociodemographic factors, such as head of household occupation (e.g. merchant, teacher, or public servant), socioeconomic characteristics, and malaria prevalence. Gender-based differences in infection rates were analysed, as well as the impact of the household head’s educational level. Socioeconomic status was examined using wealth quintiles to evaluate economic disparities in malaria infection. However, social capital was not evaluated in the present study.

### Statistical analysis

Before conducting the primary analyses, we performed a baseline comparison of children and adolescents enrolled in the Maferinyah cohort. This comparison served to contextualise key demographic, socioeconomic, and household-level characteristics between the two age groups.

Socioeconomic position (SEP) was derived using principal component analysis (PCA) of household asset variables, including items such as television, radio, land ownership, transportation means, and mobile phone ownership. Households were then grouped into low, middle, and high SEP categories based on their PCA scores. The concentration index (CI) was calculated to assess socioeconomic inequities in malaria infection. Values near zero indicate no socioeconomic gradient, whereas positive and negative values indicate a higher malaria burden among wealthier and poorer households, respectively. Next, a bivariate mixed-effects logistic regression model was employed to account for the longitudinal nature of the data and the resulting within-household correlations. Each covariate was examined individually (bivariate framework), and random intercepts at the household level were included to capture the unobserved heterogeneity. Separate analyses were performed for children and adolescents and for the dry and rainy seasons to reflect the potential variations in malaria risk across these strata.

Finally, a decomposition analysis was carried out using the *rineq package* in R to partition the overall CI into contributions from specific explanatory variables (e.g. household head’s age, sex, education, marital status, occupation, and SEP level). In this procedure, the contribution of each factor was as follows:

- Elasticity, which indicates how changes in that factor relate to changes in malaria risk,
- The variable’s own concentration index, describing its distribution across wealth levels,
- The absolute contribution (Contribution (Abs)), reflecting each factor’s direct share of the total inequity, and.
- The percentage contribution (contribution (%)), which indicates the proportion of the overall CI explained by that factor relative to the other variables. These parameters were computed separately for dry and rainy seasons, yielding columns such as “Contribution (%) (Dry),” “Contribution (%) (Rainy),” “Contribution (Abs) (Dry),” “Contribution (Abs) (Rainy),” “Elasticity (Dry),” “Elasticity (Rainy),” “Concentration Index (Dry),” and “Concentration Index (Rainy).”

All analyses were performed using R (version 4.3.3). Statistical significance was set at *P* < 0.05.

## Results

### Flowchart of inclusion

**Fig. 1:**
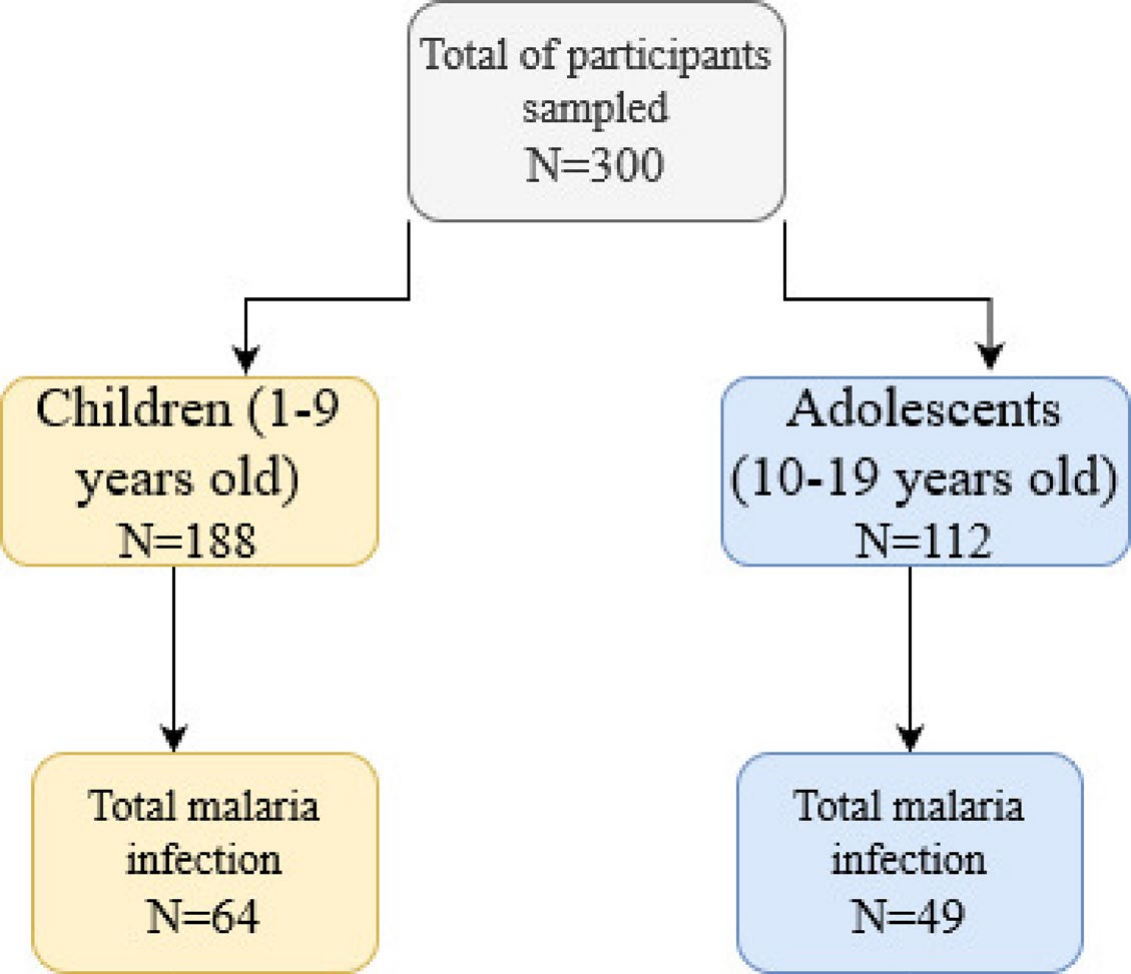
Flowchart of malaria infection in children and adolescents. A longitudinal study in Maferinyah, Guinea.

Figure 1 illustrates the distribution of malaria infection among children and adolescents in a rural area of the Republic of Guinea. Of the 300 participants sampled, 188 were children aged 1–9 years, with 64 malaria infections during the follow-up period. The remaining 112 participants were adolescents aged 10–19 years, 49 of whom were infected.

### Participant characteristics at baseline

Table 1 (Additional File 1) shows the recruitment status of the recruited adolescents and children. At recruitment, adolescents and children in the Maferinyah cohort were broadly similar across a wide range of socio-demographic and household characteristics. No statistically significant differences were observed in sex distribution, head of household age, sex, education, marital status, occupation, household size, media access (TV, Internet), insecticide-treated net (ITN) access and use, mosquito repellent use, recent travel, parasitaemia density, or recruitment season. However, modest but statistically significant differences were noted in residence (*p* = 0.029), where more children lived in area 1; radio access (*p* = 0.028), with children reporting higher access; and wealth distribution (*p* = 0.038), where adolescents were more frequently in the low socioeconomic group. Additionally, the prevalence of malaria infection was slightly higher among adolescents than among children (7.9% vs. 5.0%, *p* = 0.005). These differences were limited, and the baseline characteristics of the two groups were comparable.

**Table 1:** Concentration index of malaria infection among children and adolescents by season. Maferinyah, 2023.

Season and group	Concentration Index [95% CI]
Rainy season (children)	0.128 [− 0.069, 0.325]
Dry season (children)	− 0.019 [− 0.19, 0.152]
Rainy season (adolescents)	0.085 [− 0.094, 0.265]
Dry season (adolescents)	0.059 [− 0.121, 0.24]

### Concentration index for children and adolescents according to season (dry and rainy)

Table 1 presents the concentration indices for malaria infection among children and adolescents across different seasons in Maferinyah in 2023. The concentration index reveals patterns of socioeconomic inequity in the distribution of malaria. For children during the rainy season, the concentration index was 0.128 (95% CI -0.069, 0.325), suggesting a tendency toward higher malaria concentration among wealthier households, whereas during the dry season, a slightly negative value of -0.019 (95% CI -0.19, 0.152) was observed, indicating minimal inequity with a slight tendency toward concentration among poorer households. Among adolescents, both seasons showed positive concentration indices (rainy season: 0.085, 95% CI -0.094–0.265; dry season: 0.059, 95% CI -0.121–0.24), suggesting a modest but non-significant trend toward higher malaria prevalence in wealthier households than in poorer households.

### Bivariate mixed logistic regression analysis. Malaria infection among children and adolescents according to season and sociodemographic characteristics

Table 2 presents the distribution of malaria among children and adolescents across different seasons in Maferinyah in 2023. Among children during the dry season, residence in households headed by older adults (50–77 years) was associated with significantly elevated malaria risk (OR = 3.44, 95% CI 1.03–11.49, *p* = 0.044) compared with younger household heads (18–34 years). Similarly, children residing in Area 2 exhibited significantly higher malaria odds during the dry season than those in Area 1 (OR = 2.85, 95% CI 1.08–7.56, *p* = 0.035). The vulnerability pattern shifted notably for adolescents during the rainy season, with dramatically increased odds observed among those living with middle-aged household heads (35–49 years) (OR = 15.78, 95% CI 2.7-92.29, *p* = 0.002) and in households with a single marital status (OR = 4.52, 95% CI 1.13–18.04, *p* = 0.033).

**Table 2:** Malaria infection among children and adolescents according to the season. Bivariate mixed logistic regression analysis. Maferinyah, 2023.

Characteristics	Children				Adolescents			
	OR (rainy season, children) [95% CI]	*p*-value (rainy season, children) [95% CI]	OR (dry season, children) [95% CI]	*p*-value (dry season, children) [95% CI]	OR (rainy season, adolescents) [95% CI]	*p*-value (rainy season, adolescents) [95% CI]	OR (dry season, adolescents) [95% CI]	*p*-value (dry season, adolescents) [95% CI]
Head household age (ref.=18–34)								
Head household age [35,49]	1.19 [0.2, 6.96]	0.847	0.89 [0.22, 3.54]	0.871	15.78 [2.7, 92.29]	0.002	1.67 [0.19, 14.57]	0.641
Head household age [50,77]	1.11 [0.2, 6.23]	0.904	3.44 [1.03, 11.49]	0.044	2.42 [0.43, 13.56]	0.315	2.24 [0.26, 19.39]	0.463
Head household education (ref.=No formal education)								
Head household education (Primary)	0.55 [0.1, 3.03]	0.493	0.49 [0.09, 2.55]	0.393	0.92 [0.22, 3.85]	0.908	1.26 [0.23, 7.04]	0.788
Head household education (Secondary)	0.45 [0.14, 1.41]	0.171	0.58 [0.21, 1.66]	0.314	0.84 [0.3, 2.4]	0.749	0.28 [0.06, 1.21]	0.089
Head household gender (ref.= female)								
Male headed household	3.08 [0.97, 9.78]	0.056	0.57 [0.19, 1.66]	0.302	1.87 [0.72, 4.91]	0.201	1.35 [0.25, 7.33]	0.728
Marital status (ref.= married)								
Head’s marital status (Single)	0.68 [0.05, 9.97]	0.780	1.53 [0.3, 7.94]	0.612	4.52 [1.13, 18.04]	0.033	1.69 [0.09, 32.74]	0.727
Head household occupation (Ref.= farmer								
Head household occupation (Merchant)	0.71 [0.08, 6.16]	0.757	0.27 [0.03, 2.29]	0.231	1.99 [0.26, 15.11]	0.505	1.47 [0.12, 18.47]	0.765
Head household occupation (Public Servant)	0.68 [0.08, 5.61]	0.721	0.67 [0.11, 4.28]	0.674	7.89 [0.94, 66.45]	0.057	0 [0, Inf]	0.995
Head household occupation (Unemployed)	2.06 [0.37, 11.29]	0.407	1.06 [0.24, 4.77]	0.940	0.81 [0.12, 5.29]	0.827	1.12 [0.11, 11.39]	0.922
Residence (ref.= area 1)								
Residence (area 2)	1.7 [0.49, 5.89]	0.405	2.85 [1.08, 7.56]	0.035	1.06 [0.4, 2.8]	0.910	2.05 [0.37, 11.22]	0.408

### Decomposition analysis of the concentration index of malaria infection among children

Table 3 presents a comprehensive decomposition analysis of the malaria concentration index for children in Maferinyah (2023). Secondary education of household heads emerged as the dominant positive contributor (246.811% in the dry season, 49.168% in the rainy season) and elasticity (− 0.036, − 0.014), indicating an inequity effect that concentrates malaria among poor households. Conversely, primary education (dry years: 88.880%; rainy years: -32.995%) demonstrated substantial inequity-increasing effects. The occupational variables reveal important nuances, with merchant occupations showing a negative contribution (-83.216% in the dry season). Geographic factors were equally critical, with residence in Area 2 contributing significantly to inequity reduction during the dry season (-68.576%) but minimally during the rainy season (− 5.835%). Notably, the age of the household head was significantly negatively associated with inequity, particularly among those aged 50–77 years (− 95.3% in the dry season).

**Table 3:** Decomposition analysis of the malaria concentration index for children. Maferinyah, 2023.

	Contribution (%) (dry)	Contribution (%) (rainy)	Contribution (abs) (dry)	Contribution (abs) (rainy)	Elasticity (dry)	Elasticity (rainy)	Concentration index (dry)	Concentration Index (rainy)
Residence (ref.= area 1)								
Area 2	− 68.576	− 5.835	0.007	0.003	0.012	− 0.013	0.256	0.296
Head household age (ref.=18–34)								
Head household age [35,49]	− 31.957	6.413	0.003	− 0.004	− 0.038	− 0.077	− 0.248	− 0.280
Head household age [50,77]	− 95.343	− 36.586	0.009	0.022	0.034	− 0.235	0.006	0.013
Head household gender (ref.= female)								
Male headed household	− 1.955	5.238	0.000	− 0.003	0.034	0.073	0.256	0.266
Head household education (ref.= no formal education)								
Head household education (primary)	− 88.880	− 32.995	0.009	0.019	0.144	0.159	− 0.170	− 0.183
Head household education (secondary)	246.811	49.168	− 0.025	− 0.029	− 0.036	− 0.014	0.110	0.117
Marital status (ref.= married)								
Head’s marital status (single)	39.912	2.835	− 0.004	− 0.002	0.045	0.033	0.185	0.213
Head household occupation (Ref.= farmer)								
Head household occupation (merchant)	− 83.216	− 11.757	0.008	0.007	0.038	− 0.003	0.002	− 0.013
Head household occupation (public servant)	− 0.592	− 0.056	0.000	0.000	− 0.038	− 0.186	− 0.036	− 0.037
Head household occupation (unemployed)	− 13.715	− 11.820	0.001	0.007	0.016	0.031	− 0.082	− 0.089

In this study, the age of the household head, particularly those aged 50–77 years, was significantly associated with a reduction in malaria-related inequity, contributing up to − 95.3% during the dry season. While this effect is context-specific and should not be interpreted as universally protective, it suggests that in this setting, older household heads may have provided more equitable malaria-related support across socioeconomic strata, potentially due to their accumulated experience or household stability.

### Decomposition analysis of the concentration of malaria infection among adolescents

Table 4 presents a detailed decomposition analysis of the malaria concentration index for adolescents in Maferinyah (2023). Primary education of household heads shows a substantial positive contribution (311.643%) during the dry season but shifts to a negative contribution (− 15.676%) during the rainy season. Similarly, residence in Area 2 exhibited a strong positive contribution (157.418%) during the dry season but a minimal negative contribution (− 4.110%) during rainy periods. The age of household heads presented contrasting patterns, with middle− aged heads (35–49) contributing positively to inequity in both seasons (86.348% dry, 144.439% rainy), while older household heads (50–77) show a substantial negative contribution (− 419.277%) during the dry season. Unemployment among household heads demonstrated a significant negative contribution (− 184.240%) during the dry season, and the elasticity values revealed important insights into inequity mechanisms. The results indicated that Male-headed households contributed − 67.186% to malaria-related health inequity among adolescents during the dry season and + 19.494% during the rainy season, suggesting a seasonal reversal in the direction of their contribution. The absolute contributions were 0.003 and 0.007 in the dry and rainy seasons, respectively, indicating a greater impact in the latter season. The concentration index was 0.337 during the dry season and 0.340 during the rainy season, indicating that male-headed households were consistently concentrated in socioeconomically advantaged groups in both seasons.

**Table 4:** Decomposition analysis of the malaria concentration index for adolescents. Maferinyah, 2023.

Characteristics	Contribution (%) (Dry)	Contribution (%) (Rainy)	Contribution (Abs) (Dry)	Contribution (Abs) (Rainy)	Elasticity (Dry)	Elasticity (Rainy)	Concentration Index (Dry)	Concentration Index (Rainy)
Residence (ref.= Area 1)								
Area 2	157.418	− 4.110	− 0.008	− 0.001	− 0.033	0.408	0.129	0.126
Head household age (ref.=18–34)								
Head household age [35,49]	86.348	144.439	− 0.004	0.051	− 0.130	0.068	− 0.159	− 0.181
Head household age [50,77]	− 419.277	− 34.713	0.021	− 0.012	0.088	0.143	0.038	0.048
Head household gender (ref.= female)								
Male headed household	− 67.186	19.494	0.003	0.007	− 0.046	− 0.016	0.337	0.340
Head household education (ref.= no formal education)								
Head household education (primary)	311.643	− 15.676	− 0.015	− 0.006	0.019	− 0.192	− 0.179	− 0.148
Head household education (secondary)	67.643	79.748	− 0.003	0.028	− 0.030	0.070	0.163	0.197
Marital status (ref.= married)								
Head’s marital status (single)	99.097	38.820	− 0.005	0.014	− 0.049	− 0.176	0.061	0.072
Head household occupation (Ref.= Farmer								
Head household occupation (merchant)	59.341	− 35.707	− 0.003	− 0.013	0.024	0.005	− 0.035	− 0.122
Head household occupation (public servant)	16.947	− 1.742	− 0.001	− 0.001	− 0.205	− 0.321	− 0.044	− 0.009
Head household occupation (unemployed)	− 184.240	7.723	0.009	0.003	0.086	− 0.062	− 0.282	− 0.262

## Discussion

In this study, we examined seasonal variations in the equity-related drivers of malaria transmission among children and adolescents in rural areas of Guinea. The results indicate that the burden of malaria is not only inequitably distributed but is also modulated by seasonal changes, with socioeconomic status, household head’s education, and household structure emerging as significant determinants of malaria prevalence.

Several household-level determinants emerged as key contributors to socioeconomic health inequity, with distinct seasonal variations observed between the dry and rainy periods of the study. Notably, the age of the head of household, particularly among those aged 50–77 years, contributed significantly and negatively to malaria-related inequity during the dry season (typically February to May, which falls outside the main harvest period). During this time, households often experience resource depletion due to the limited storage of agricultural yields, which exacerbates their vulnerabilities^33^. The negative contribution to inequity indicates that older-headed households were more concentrated among wealthier groups; thus, their heightened malaria risk during the resource-scarce dry season disproportionately affected the higher socioeconomic strata.

Further developing this, household heads aged 59–76 contribute negatively to reducing the concentration index, indicating a lower prevalence of malaria among disadvantaged groups with older heads. The age of household heads may influence malaria transmission among children and adolescents through various mechanisms, including knowledge and practices related to malaria prevention^34^, economic stability, and contextual factors. This result may reflect contextual dynamics specific to rural areas like Maferinyah, where older individuals often possess greater social capital, accumulated experience, and economic stability, which can enable more effective implementation of preventive measures. Additionally, older household heads may have more time or willingness to engage in malaria prevention, especially during the dry season, when agricultural activities are reduced. In contrast, younger heads may face competing demands due to occupational mobility or limited household authority, potentially constraining consistent malaria prevention efforts. These findings underscore how age intersects with household roles and socioeconomic factors to shape vulnerability in a complex manner.

The education level of household heads, particularly secondary education, was associated with a reduction in malaria-related inequity. Households with heads who had attained secondary education were generally wealthier and better protected, and higher education attainment likely translated into improved awareness and uptake of malaria prevention measures. This finding highlights the important role of educational attainment as a structural determinant of health, reinforcing the notion that investments in household head education can contribute to narrowing social disparities in malaria risk. The influence of household head education on malaria transmission among children and adolescents appears to be both protective and equity-enhancing in this context. Consistent with the previous literature^35–38^, studies have indicated that the higher the educational attainment of the household head, the lower the risk of malaria infection among children and adolescents. Similarly, in sub-Saharan Africa, six years of maternal schooling was significantly associated with lower odds of malaria infection in children^35^. Moreover, in Western Kenya, mother’s education level is an important factor in reducing the risk of clinical malaria infection^36^. Another study found that children whose mothers had education beyond primary school were 4.7% less likely o be malaria positive^37^. The educational level of household heads has broader implications, influencing community practices and the overall malaria transmission rate. Higher education levels in household heads correlate with increased community awareness and the use of preventive measures^38^. Educated parents are more likely to implement and advocate preventive measures such as using insecticide-treated nets, ensuring prompt medical attention, and supporting public health campaigns. For instance, one study found that " efforts should be made to educate school children on the importance of malaria prevention as well as ensuring that household members are well-informed“^39^.

Moreover, our study revealed the influence of socioeconomic position on malaria transmission and that socioeconomic status (SES) plays a significant role in malaria transmission among children and adolescents. Various studies have shown that a lower socioeconomic status is associated with higher malaria prevalence^40,41^. For instance, in Cameroon, children from poorer households had a malaria prevalence of 33.8%, whic decreased to 13.3% amongchildren from wealthier households, highlighting the impact of household well-being on malaria infection rates^40^. SES also influences access to healthcare facilities and the likelihood of receiving a malaria diagnosis and treatment. In sub-Saharan Africa, children from lower wealth quintiles were less likely to be taken to medical facilities and receive blood tests than those from higher wealth quintiles^42^. Overall, socioeconomic factors, such as household wealth and environmental^43^ conditions, significantly influence malaria transmission.

By exploring gender-related factors, we observed seasonal variations in the contribution of male-headed households to malaria-related health inequity in adolescents. During the dry season, male-headed households were associated with a reduction in malaria-related inequity, suggesting a more equitable distribution of malaria in these households than in their female counterparts. Male-headed households contribute differently to the concentration index, with minimal impact on overall inequity. Male-headed households (MHHs) may have different health outcomes for children, potentially influenced by varying priorities and practices within the household^44^. Female-headed households (FHHs) are more likely to engage in preventive measures, such as spraying insecticides and maintaining clean surroundings, whereas MHHs are more likely to own insecticide-treated bed nets^45^. These preventive measures directly affect children’s exposure to malaria vectors, thereby influencing transmission rates.

In the context of Maferinyah, the heightened inequity in malaria risk among female-headed households during the dry season may be explained by multiple structural and social factors. Female-headed households in rural West Africa often face lower incomes, limited access to prevention tools (such as bed nets), and higher unpaid caregiving burdens, particularly during periods of economic scarcity^33,46^.

In contrast, during the rainy season, male-headed households positively contributed to malaria-related inequity. This shift may be partially explained by changes in adolescent routines during the rainy season, particularly in rural settings such as Maferinyah. Adolescent boys in male-headed households are often engaged in labor-intensive or mobile economic activities, such as farming or small-scale trading, or participate in late-night social activities, such as football viewing or video halls, which increase their outdoor exposure during peak Anopheles biting hours^47^. Adolescent girls, while less mobile, also face increased exposure during this period through household tasks like early morning or evening cooking, typically performed outdoors^48,49^. These gendered behavioural patterns may contribute to higher exposure among adolescents.

Subsequently, place of residence emerged as a significant contributor to malaria-related health inequities, with Area 2 demonstrating contrasting effects across age groups during the dry season. Specifically, Area 2 was associated with a reduction in malaria inequity among children but an increase among adolescents. This area benefits from relatively better infrastructure, including well-constructed housing and improved sanitation. Notably, the recent establishment of training institutions for nurses and community health workers in Area 2 has enhanced local malaria prevention and control efforts, with students actively participating in community outreach. Additionally, the presence of our research centre in this area has ensured more comprehensive malaria-related services for the local population. However, the increased inequity observed among adolescents may reflect gaps in targeted health services. Adolescents are often overlooked in diagnostic and treatment efforts and are generally expected to manage their own health, which can lead to delayed care-seeking and under-diagnosis.

Furthermore, Occupational status reveals important nuances in its contribution to malaria-related health inequity. For example, merchant occupations showed a substantial negative contribution to inequity during the dry season (-83.216%), indicating that this group was associated with a reduction in socioeconomic inequity in malaria infection. This may suggest that individuals in merchant households who are likely to be more mobile and economically active across wealth gradients may have contributed to a more equitable distribution of malaria risk during this period, possibly due to differential access to preventive tools. These findings underscore the relevance of occupation not only as a socioeconomic marker but also as a determinant of exposure patterns that shape the distribution of the malaria burden.

Conversely, the situation for adolescents is notably different. Merchant households contributed positively to inequity in malaria infection among adolescents. This may be attributed to adolescents often assisting their parents in trading activities, which increases their mobility and outdoor exposure, particularly during periods of peak mosquito activity. A study in Taraba State, Nigeria, found that traders had the highest malaria prevalence (86%)^50^.

In contrast, unemployment showed a negative contribution to inequity and a negative concentration index among adolescents, indicating a higher malaria burden in disadvantaged households. This aligns with the findings that unemployment, often linked to poverty and restricted access to healthcare, can undermine a household’s capacity to afford preventive measures or seek timely treatment^28^. For instance, a study in Kenya found that children from poor households had a higher prevalence of malaria^51^. In contrast, our study found that employment contributed to increased inequity among adolescents, suggesting that work-related mobility or other employment-related factors may disproportionately increase malaria risk in this group. Adolescents living in households headed by unemployed individuals may be more aware of their families’ socioeconomic challenges and, as a result, more proactive in seeking care. This is particularly relevant in the context of our study, where participants received free malaria treatment, which may have encouraged timely care-seeking behaviours, regardless of household employment status.

Finally, we found that single-headed households contribute positively to inequity, indicating a greater prevalence of malaria among the advantaged households. This result aligns with findings from rural Nigerian settings, where non-cohabiting parents were identified as significant predictors of severe malaria among children under five years of age, suggesting that family structure affects malaria susceptibility^52^. This may be due to a lack of resources and supervision in single-headed households, which can limit access to preventive measures such as insecticide-treated nets (ITNs). Single-headed households, particularly those headed by women, often face economic hardships that contribute to poor living conditions. For example, a study in Khartoum State, Sudan, highlighted that such households are more likely to live in poor environmental conditions, which increases the risk of malaria transmission^43^. Although previous findings have indicated a pro-rich distribution of malaria among single-headed households, this does not necessarily reflect equitable health outcomes for all members. In this context, wealthier single-headed households may still face an elevated malaria risk because of time constraints and limited caregiving capacity. Despite their relative economic advantage, single parents often engage in demanding income-generating activities and may have less time to supervise their children or ensure the consistent use of preventive measures such as bed nets. For instance, in Maferinyah, a major local marketplace that attracts traders from surrounding villages every weekend, single-headed households are frequently involved in commercial activities that require their presence, leaving children more exposed and less supervised during peak mosquito hours.

### Strengths and limitations

This is one of the first studies to identify the factors contributing to inequities in malaria transmission within a rural setting, exploring the potential to mitigate these disparities through sociodemographic and socioeconomic factors, using the PROGRESS framework. Although this study included numerous relevant factors, providing a solid foundation for meaningful conclusions, there are some limitations to consider. The presence of extremely high contributions (positive or negative) may reflect the strong influence of certain factors, such as “Residence (Area 2)”, and points to the complexity of the determinants influencing malaria inequity. Further research should explore the underlying mechanisms of these factors to provide greater clarity and refine intervention strategies. Finally, the reduction in malaria cases over time may have been influenced by monthly visits by the research team, highlighting the need for routine malaria surveillance over an extended period to validate and strengthen these findings in the future.

## Conclusion

This study highlights the critical inequities in malaria transmission among children and adolescents in rural Guinea, shaped by social determinants such as household structure, education, socioeconomic status, occupation, and age of household heads. Disadvantaged groups, particularly female- or single-headed households, those with lower educational attainment, and households engaged in unstable or informal work, experienced a disproportionately higher malaria burden, especially during the rainy season. Notably, older household heads were associated with reduced malaria prevalence, possibly because of their greater experience managing household health.

To effectively address these inequities, malaria control strategies must move beyond universal approaches and embrace seasonally timed, socially responsive, and equity-enhancing interventions. The key recommendations are as follows:

- Targeted indoor residual spraying (IRS) and long-lasting insecticidal net (LLINA) distribution, prioritising female-headed or occupationally vulnerable households.
- Community-based health education campaigns tailored to the roles and risk behaviours of different household members, such as adolescents’ evening mobility and women’s domestic exposure during peak mosquito-biting hours.
- Flexible service delivery models, including mobile health teams deployed during the rainy season, are used to reach remote or structurally disadvantaged households.
- Expanding seasonal malaria chemoprevention (SMC) to include older children and adolescents based on their age-specific risk patterns.
- Promoting complementary prevention tools for those facing outdoor occupational exposure (e.g. farmers and traders), such as spatial repellents, insecticide-treated clothing, and protective practices during early evening hours.

Aligning malaria control strategies with household composition, occupational dynamics, and seasonal vulnerability patterns is essential not only for improving program effectiveness but also for promoting greater health equity in malaria-endemic settings.

## Supplementary Information

Below is the link to the electronic supplementary material.

Supplementary Material 1.

Supplementary Material 2.

Supplementary Material 3.

Supplementary Material 4.

Supplementary Material 5.

Supplementary Material 6.

Supplementary Material 7.

Supplementary Material 8.

Supplementary Material 9.

Supplementary Material 10.

## Data Availability

Availability of data and materialsThe dataset supporting the conclusions of this study is included within the article and its additional files 2, 3, 4, 5, 6,7, 7,8 and 9.The survey and questionnaire form were included within the article and its additional files 10.

## Abbreviations

OR: Adjusted odds ratio
CI: Confidence interval
