# Supplementary material for: Inequity in the transmission of malaria infection among children and adolescents: a cohort study in rural Guinea
Source: Sci Rep. 2025 Aug 14;15:29881. doi: 10.1038/s41598-025-15600-w (PMC12354881; doi:10.1038/s41598-025-15600-w)
Supplement: Supplementary file 1 — Supplementary Material 1. [file 41598_2025_15600_MOESM1_ESM.docx]

**Inequity in the transmission of malaria infection among children and adolescents: A cohort study in a rural area, Guinea.**

**Authors’ affiliations**

Almamy Amara TOURE^1,2¥^, Sidikiba SIDIBE^2,6^, Aboubacar Sidiki MAGASSOUBA^2^, Abdoul Habib BEAVOGUI^3^, Mamoudou CONDE^4^, Abdoulaye Fodé TOURE^4^, Tiany SIDIBE^5^, Kaba Saran KEITA^5^, Alexandre DELAMOU^2,6^, Seni KOUANDA^1,7^

1.Institut Africain de Santé Publique (IASP/USTA) of the University Saint Thomas D’Aquin, Ouagadougou, Burkina Faso. 2. Department of Public Health, Faculty of Health Sciences and Techniques, Gamal Abdel Nasser University, Conakry, Guinea. 3. Centre National de Formation et de Recherche en Santé Rurale de Mafèrinyah, Forécariah, Guinea. 4. National Institute of Public Health, Coyah, Guinea. 5. Department of Public Health, Center for Research in Reproductive Health in Guinea, Conakry, Guinea 6. Centre d´Excellence Africain pour la Prévention et le Contrôle des Maladies Transmissibles (CEA-PCMT), Gamal Abdel Nasser University, Conakry, Guinea. 7. Institut Africain de Santé publique, Ouagadougou, Burkina Faso.

**Authors' email address**

Almamy Amara TOURE : [almamy@maferinyah.org](mailto:almamy@maferinyah.org)

Sidikiba SIDIBE : [layesidikiba@gmail.com](mailto:layesidikiba@gmail.com)

Aboubacar Sidiki MAGASSOUBA : [magasbakary01@yahoo.fr](mailto:magasbakary01@yahoo.fr)

Abdoul Habib BEAVOGUI: [bea@maferinyah.org](mailto:bea@maferinyah.org)

Abdoulaye Fode TOURE : [abdoulayefodetoure@gmail.com](mailto:abdoulayefodetoure@gmail.com)

Mamoudou CONDE : [mamoudouconde89@gmail.com](mailto:mamoudouconde89@gmail.com)

Tiany SIDIBE : [sidibetiany@gmail.com](mailto:sidibetiany@gmail.com)

Kaba Saran KEITA : [kabassan85@gmail.com](mailto:kabassan85@gmail.com)

Alexandre DELAMOU : [adelamou@cea-pcmt.org](mailto:adelamou@cea-pcmt.org)

Seni KOUANDA: [senikouanda@gmail.com](mailto:senikouanda@gmail.com)

**Supplementary**

**Table 1: Baseline comparison between adolescents and children. Maferinyah, cohort study.**

|  | **Adolescents** | **Children** | **p.overall** |
| --- | --- | --- | --- |
|  | ***N=109*** | ***N=188*** |  |
| Gender |  |  | 0.366 |
| Female | 51.38% [41.61%;61.06%] | 45.21% [37.96%;52.62%] |  |
| Male | 48.62% [38.94%;58.39%] | 54.79% [47.38%;62.04%] |  |
| residence: |  |  | 0.029 |
| area1 | 36.70% [27.67%;46.47%] | 50.53% [43.16%;57.89%] |  |
| area2 | 63.30% [53.53%;72.33%] | 49.47% [42.11%;56.84%] |  |
| Head of household age |  |  | 0.655 |
| Less than 35 | 25.69% [17.80%;34.94%] | 29.26% [22.86%;36.32%] |  |
| [36,50] | 38.53% [29.37%;48.34%] | 33.51% [26.81%;40.74%] |  |
| More than 50 | 35.78% [26.83%;45.53%] | 37.23% [30.31%;44.57%] |  |
| Head of household gender |  |  | 0.819 |
| Men | 43.12% [33.67%;52.95%] | 45.21% [37.96%;52.62%] |  |
| Women | 56.88% [47.05%;66.33%] | 54.79% [47.38%;62.04%] |  |
| Head of household education |  |  | 0.608 |
| No formal education | 31.19% [22.66%;40.78%] | 31.38% [24.83%;38.54%] |  |
| Primary | 17.43% [10.83%;25.87%] | 13.30% [8.79%;19.00%] |  |
| Secondary | 51.38% [41.61%;61.06%] | 55.32% [47.91%;62.56%] |  |
| Head of household marital status |  |  | 0.986 |
| Married | 91.74% [84.90%;96.15%] | 90.96% [85.92%;94.64%] |  |
| Single | 8.26% [3.85%;15.10%] | 9.04% [5.36%;14.08%] |  |
| Head of household occupation |  |  | 0.298 |
| Farmer | 11.01% [5.82%;18.44%] | 14.89% [10.13%;20.80%] |  |
| Merchant | 22.02% [14.65%;30.97%] | 17.02% [11.94%;23.17%] |  |
| Civil servant | 11.01% [5.82%;18.44%] | 17.02% [11.94%;23.17%] |  |
| Unemployed | 55.96% [46.13%;65.46%] | 51.06% [43.69%;58.41%] |  |
| Access radio |  |  | 0.028 |
| No access | 55.96% [46.13%;65.46%] | 42.02% [34.88%;49.42%] |  |
| Access | 44.04% [34.54%;53.87%] | 57.98% [50.58%;65.12%] |  |
| TV access |  |  | 0.538 |
| No access | 28.44% [20.21%;37.88%] | 24.47% [18.50%;31.25%] |  |
| Access | 71.56% [62.12%;79.79%] | 75.53% [68.75%;81.50%] |  |
| Internet |  |  | 1.000 |
| No access | 52.29% [42.51%;61.95%] | 52.66% [45.26%;59.97%] |  |
| Access | 47.71% [38.05%;57.49%] | 47.34% [40.03%;54.74%] |  |
| Insecticide Treated Net access |  |  | 0.615 |
| No | 40.37% [31.08%;50.19%] | 36.70% [29.81%;44.02%] |  |
| Yes | 59.63% [49.81%;68.92%] | 63.30% [55.98%;70.19%] |  |
| Use of ITN |  |  | 0.296 |
| No | 49.54% [39.82%;59.28%] | 42.55% [35.39%;49.96%] |  |
| Yes | 50.46% [40.72%;60.18%] | 57.45% [50.04%;64.61%] |  |
| Has child travelled far from study setting? |  |  | 0.270 |
| No | 97.25% [92.17%;99.43%] | 93.62% [89.12%;96.66%] |  |
| Yes | 2.75% [0.57%;7.83%] | 6.38% [3.34%;10.88%] |  |
| Use of mosquito repellent |  |  | 0.720 |
| No | 41.28% [31.94%;51.12%] | 44.15% [36.93%;51.56%] |  |
| Yes | 58.72% [48.88%;68.06%] | 55.85% [48.44%;63.07%] |  |
| Number of household members |  |  | 0.695 |
| [3,5] | 21.10% [13.87%;29.96%] | 22.34% [16.60%;28.97%] |  |
| [6,12] | 59.63% [49.81%;68.92%] | 62.23% [54.89%;69.19%] |  |
| [13,35] | 19.27% [12.34%;27.93%] | 15.43% [10.58%;21.40%] |  |
| Density of parasitemia | 353.13 [-235.28;941.54] | 422.10 [-103.15;947.36] | 0.863 |
| Wealth quintile |  |  | 0.038 |
| Low socioeconomic position (SEP) | 39.45% [30.22%;49.27%] | 27.13% [20.91%;34.08%] |  |
| Middle_SEP | 26.61% [18.60%;35.93%] | 39.36% [32.33%;46.73%] |  |
| High SEP | 33.94% [25.15%;43.64%] | 33.51% [26.81%;40.74%] |  |
| Malaria infection |  |  | 0.005 |
| Negative | 92.06% [90.19%;93.67%] | 94.97% [93.71%;96.05%] |  |
| Positive | 7.94% [6.33%;9.81%] | 5.03% [3.95%;6.29%] |  |
| Season |  |  | 0.555 |
| Dry season | 57.03% [53.86%;60.15%] | 55.73% [53.11%;58.32%] |  |
| Rainy season | 42.97% [39.85%;46.14%] | 44.27% [41.68%;46.89%] |  |
